# Accelerated hyperfractionation (AHF) compared to conventional fractionation (CF) in the postoperative radiotherapy of locally advanced head and neck cancer: influence of proliferation

**DOI:** 10.1038/sj.bjc.6600119

**Published:** 2002-02-12

**Authors:** H K Awwad, M Lotayef, T Shouman, A C Begg, G Wilson, S M Bentzen, H Abd El-Moneim, S Eissa

**Affiliations:** Department of Radiotherapy, National Cancer Institute, University of Cairo, Fom El Khalig 11796, Cairo, Egypt; Division of Experimental Therapy, The Netherlands Cancer Institute, Plesmanlaan 121, 1066 Amsterdam, The Netherlands; Experimental Oncology, Gray Cancer Institute, Northwood, Middlesex 2JR, UK; Biostatistics in Oncology, Gray Cancer Institute, Northwood, Middlesex 2JR, UK

**Keywords:** head and neck cancer, postoperative radiotherapy, accelerated hyperfractionation, proliferation kinetics, predictive factors

## Abstract

Based on the assumption that an accelerated proliferation process prevails in tumour cell residues after surgery, the possibility that treatment acceleration would offer a therapeutic advantage in postoperative radiotherapy of locally advanced head and neck cancer was investigated. The value of T_pot_ in predicting the treatment outcome and in selecting patients for accelerated fractionation was tested. Seventy patients with (T2/N1–N2) or (T3-4/any N) squamous cell carcinoma of the oral cavity, larynx and hypopharynx who underwent radical surgery, were randomized to either (a) accelerated hyperfractionation: 46.2 Gy per 12 days, 1.4 Gy per fraction, three fractions per day with 6 h interfraction interval, treating 6 days per week or (b) Conventional fractionation: 60 Gy per 6 weeks, 2 Gy per fraction, treating 5 days per week. The 3-year locoregional control rate was significantly better in the accelerated hyperfractionation (88±4%) than in the CF (57±9%) group, *P*=0.01 (and this was confirmed by multivariate analysis), but the difference in survival (60±10% *vs* 46±9%) was not significant (*P*=0.29). The favourable influence of a short treatment time was further substantiated by demonstrating the importance of the gap between surgery and radiotherapy and the overall treatment time between surgery and end of radiotherapy. Early mucositis progressed more rapidly and was more severe in the accelerated hyperfractionation group; reflecting a faster rate of dose accumulation. Xerostomia was experienced by all patients with a tendency to be more severe after accelerated hyperfractionation. Fibrosis and oedema also tended to be more frequent after accelerated hyperfractionation and probably represent consequential reactions. T_pot_ showed a correlation with disease-free survival in a univariate analysis but did not prove to be an independent factor. Moreover, the use of the minimum and corrected *P*-values did not identify a significant cut-off. Compared to conventional fractionation, accelerated hyperfractionation did not seem to offer a survival advantage in fast tumours though a better local control rate was noted. This limits the use of T_pot_ as a guide for selecting patients for accelerated hyperfractionation. For slowly growing tumours, tumour control and survival probabilities were not significantly different in the conventional fractionation and accelerated hyperfractionation groups. A rapid tumour growth was associated with a higher risk of distant metastases (*P*=0.01). In conclusion, tumour cell repopulation seems to be an important determinant of postoperative radiotherapy of locally advanced head and neck cancer despite lack of a definite association between T_pot_ and treatment outcome. In fast growing tumours accelerated hyperfractionation provided an improved local control but without a survival advantage. To gain a full benefit from treatment acceleration, the surgery-radiotherapy gap and the overall treatment time should not exceed 6 and 10 weeks respectively.

*British Journal of Cancer* (2002) **86**, 517–523. DOI: 10.1038/sj/bjc/6600119 www.bjcancer.com

© 2002 Cancer Research UK

## 

Locoregional recurrence due to regrowth of tumour cell residues is the main cause of failure of surgery for locally advanced head and neck cancer (HNC). An active proliferation process is expected to prevail in tumour cell aggregates left in the surgical field in view of a small cell number, a large growth fraction and possibly also a low cell loss factor (Awwad *et al*, 1992; Peters and Withers, 1997; Ang *et al*, 2001). The present study aims at testing the possibility of improving the postoperative radiotherapy results in locally advanced HNC by applying an accelerated fractionation scheme. The study also involves measurement of T_s_, LI and T_pot_ on the basis of a combination of flow cytometric (FCM) and immunhistochemistry analysis performed on a single tumour sample obtained 4–6 h after intravenous injection of 5-iodo-2′-deoxyuridine (IdUrd). This combination provides a better estimate of T_pot_ in diploid tumours since FCM alone tends to underestimate their proliferative activity (Begg *et al*, 1985; Bennett *et al*, 1992, Wilson, 1993). In a postoperative radiotherapy setting the pretreatment T_pot_, may be assumed to be an indicator of the effective doubling time (T_eff_) prevailing during the course of postoperative radiotherapy. Accordingly the predictive power of pretreatment proliferation parameters was tested. In addition, the possibility that accelerated fractionation can be more effective than conventional fractionation in fast growing tumours was examined.

## MATERIALS AND METHODS

### Patients and tumour characteristics

Patients less than 65 years of age with (T2/N1–2) or (T3-4/any N) squamous cell carcinoma of the oral cavity, larynx and hypopharynx who underwent radical surgery were admitted to the study provided that: (a) there was no evidence of gross residual disease or distant metastases; (b) performance status score ⩽2 according to the WHO scale, 1980; (c) liver, kidney and other vital functions were within normal. The patient written consent was also required.

### Postoperative radiotherapy schedules and techniques

Postoperative radiotherapy was planned to be commenced 3–8 weeks after surgery. The sealed envelope method was used as a simple randomization procedure. Accordingly patients were assigned to one of the two following schedules:

- *Conventional fractionation (CF).* A total dose of 60 Gy was given in 30 fractions (2 Gy per fraction) in 6 weeks treating 5 days per week.
- *Accelerated hyperfractionation (AHF).* A three fractions per day regimen was adopted with an interfraction interval of 6 h at least. A dose per fraction of 1.4 Gy was used (4.2 Gy day^−1^), with a total dose of 46.2 Gy per 33 fractions per 12 days, treating 6 days per week.

Standard postoperative radiotherapy techniques (Awwad *et al*, 1992) were applied using a Philips 6 MV linear accelerator, a Varian simulator and a Multidata computer treatment planning system. Custom-made plastic shells for head and neck immobilization were applied during treatment. The dose to the primary tumour site was prescribed according to the ICRU 50 reference point, while for neck nodes the reference was set at a depth of 0.5 cm below the skin. All portals were treated during each radiation fraction. Wherever possible the spinal cord was shielded after 40–45 Gy and the volume of the irradiated parotid salivary glands minimized.

### Cell proliferation kinetics studies

Two hundred mg of IdUrd dissolved in 10 ml saline were injected intravenously as a bolus 4–6 h before start of surgery. The time of sampling used in the subsequent *relative movement* calculation was taken as the interval between drug injection and the time of ligation of the main arterial trunks. The surgical specimen was examined conjointly with the pathologist. Multiple 0.5–1 cm^3^ pieces were taken from representative non-degenerating parts of the tumour. One or more pieces were then fixed in 10% formal saline and then embedded in paraffin for immunohistochemistry (IHC). The remaining pieces were processed for FCM.

The procedures for IdUrd staining and FCM analysis used are those described by Begg *et al* (1988) and by Wilson *et al* (1988). For immunohistochemistry 5 μm sections were mounted on poly-l-lysine coated slides. One section was stained with haematoxylin and eosin while other adjacent sections were immunohistochemically stained for IdUrd (Bennett *et al*, 1992; Wilson, 1993). For cell counting, high power fields (×40 objective×10 eyepiece) were captured and labelling was scored in a minimum of 1000 cells. The average LI was calculated as the total number of IdUrd-labelled cells divided by the total number of scored tumour cells. This was then corrected for cell division taking place during the time between injection and tumour sampling. For this the FCM LI data were used as described by Begg *et al*, 1985. The duration of the S-phase (T_s_) was derived from the FCM data using the relative movement analysis (Begg *et al*, 1985). The potential doubling time (T_pot_) was then calculated using the relationship T_pot_=λ (Ts/LI), where λ was assumed to be unity.

## RESULTS

Out of 88 consecutive patients witth locally advanced HNC who survived radical surgery during the period November 1995 and October 1997, 70 patients satisfied the eligbility criteria and were admitted to the trial and randomized to either CF (39 patients) or AHF (31 patients). Five patients (four in the CF and one in the AHF group) refused to complete the prescribed treatment due to personal and social factors i.e. an overall compliance of 93%. The representation of different clinicopathological parameters did not differ significantly in the two therapeutic groups as listed in Table 1

**Table 1:**
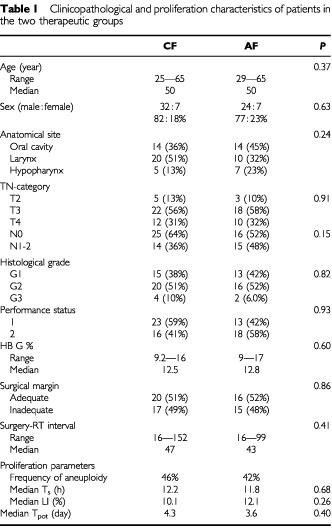
Clinicopathological and proliferation characteristics of patients in the two therapeutic groups

.

### Early normal tissue reactions

Acute skin reactions were generally mild and comparable in both therapeutic groups. In the CF-arm, acute mucositis developed gradually reaching its peak during the fourth week. In the AHF-arm, mucositis progressed rapidly from the beginning of the second week to reach its peak during the third week. In both arms complete disappearance of acute mucositis was noted by 8–10 weeks. Both objective and functional reactions tended to be significantly more severe in the AHF arm ( Table 2

**Table 2:**
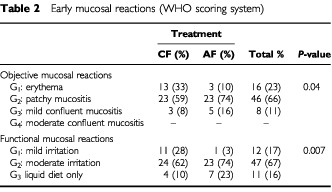
Early mucosal reactions (WHO scoring system)

). Overall, the G2 objective mucosal reaction (patchy mucositis) and G2 functional mucositis (moderate irritation) were the most common mucositis grades in both arms. Generally and in both treatment groups acute mucosal reactions did not seriously interfere with patients feeding with no weight loss nor need for tube feeding or hospitalizations for fluid or nutritional support. Simple analgesics were sufficient to relieve local pain.

### Late normal tissue reactions

All patients suffered some degree of late xerostomia (expressed as dryness of mouth and difficulty in mastication and swallowing) which persisted for 2 years at least. There was a tendency, though statistically insignificant, for xerostomia to be more severe in the AHF than in the CF group (G2 and G3 xerostomia: 33 and 42% respectively in AHF and 39 and 13% respectively in CF, *P*=0.17). The same trend was noted for G2 and three lymphoedema (CF=10%, AHF=16%) and G2 and G3 subcutaneous fibrosis (CF=13%, AHF 26%), (*P*=0.7). There was only one incidence of transient myelopathy (L'hermitte syndrome) in the AHF group which was expressed 3 months after the end of treatment and recovered within 6 months. In this patient, the dose to the cervical spinal cord was estimated to be 42 Gy.

### Loco-regional control

Loco-regional recurrence was observed in 15 out of the 70 randomized patients (three in the AHF and 12 in the CF groups). Using Kaplan–Meier (product limit) estimate, the 3-year locoregional control rate was 72±6%. Four factors were shown to influence the loco-regional control rate significantly: gender (*P*=0.04), performance status (*P*=0.01), the overall treatment time (*P*=0.005) and radiotherapy schedule (*P*=0.01) ( Table 3

**Table 3:**
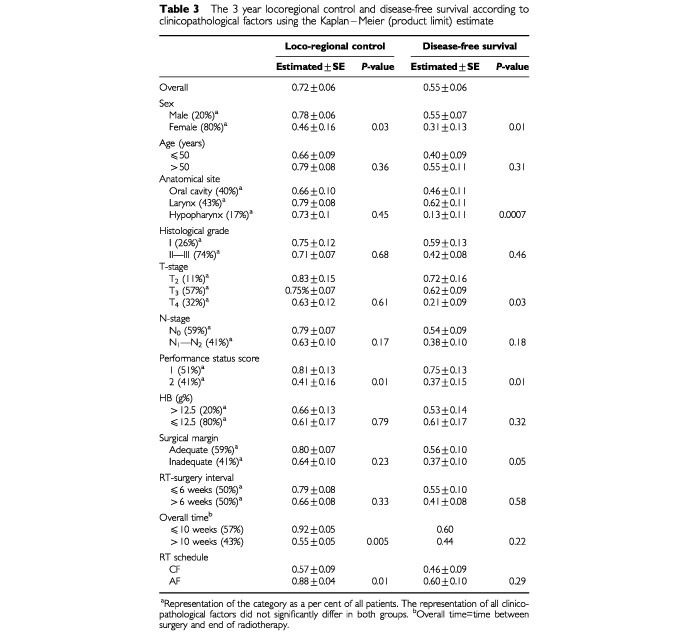
The 3 year locoregional control and disease-free survival according to clinicopathological factors using the Kaplan–Meier (product limit) estimate

). AHF was associated with a higher loco-regional control rate (88±8%) than CF (57±9%), (*P*=0.01) ( Figure 1

**Figure 1:**
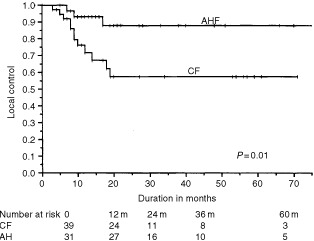
Locoregional control in the accelerated hyperfractionation (AHF) and conventional fractionation (CF) groups using the Kaplan–Meier (product limit) estimate.

). Using the Cox's proportional hazards model, the influence of the radiotherapy schedule on the loco-regional control was examined after adjusting for the distribution in the CF and AHF arms of all other variables listed in Table 3. After this adjustment, AHF remained superior to CF; the risk ratio for type of radiation treatment was 3.86 with a 95% confidence interval of 1.87 and 9.36 and a *P*-value of 0.0001.

### Survival

During the first 3 years, there were 30 deaths (17 in the CF and 13 in the AHF group) among the 70 randomized patients. According to the Kaplan–Meier estimate, the overall 3-year disease-free survival in the entire series amounted to 55±6%. The clinicopathological factors that significantly influenced survival included gender, primary site, performance status, and inadequacy of the surgical margin (Table 3). The survival rates in the AHF and CF groups did not differ significantly (*P*=0.29).

### Distant metastases

Eight patients (11%) developed distant metastases (four in each treatment group). The risk of distant metastases significantly correlated with the anatomical tumour site (*P*=0.006). Five out of the 12 patients (42%) with hypopharyngeal cancer developed metastases while the incidence was 2 out of 30 (7%) in patients with laryngeal cancer and 1 out of 28 (4%) in oral cancer. The other variables did not seem to influence the risk of metastases.

### Influence of treatment times

For the whole group, the surgery-radiotherapy interval did not seem to influence the locoregional control or the survival rates (Table 3). However, within the two fractionation groups the best *local control* result was that of the AHF when started within 6 weeks of surgery. This benefit was, however, insignificant when the treatment gap was >6 weeks ( Table 4

**Table 4:**
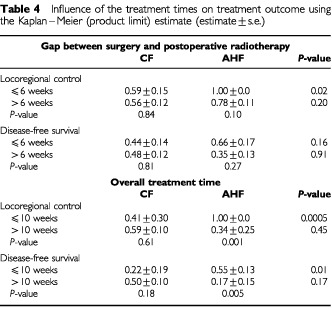
Influence of the treatment times on treatment outcome using the Kaplan–Meier (product limit) estimate (estimate±s.e.)

). Moreover, the length of the gap did not seem to influence the locoregional control results of the CF group. The gap length did not affect the survival of all patients or of patients in either treatment arm. In contrast, an overall treatment time (time between surgery and the end of radiotherapy) longer than 10 weeks had a significantly unfavourable effect on the locoregional control for all patients (Table 3) (*P*=0.005). It is interesting to note that patients in the long and short overall treatment times groups were similar in respect to their clinicopathological characteristics (data not shown). No effect was, however, observed on the disease-free survival when the entire group of patients was analyzed. However, when the AHF and CF patients were analyzed separately, the best locoregional control (*P*=0.0005) and survival (*P*=0.01) results were obtained in the AHF patients when treated over 10 weeks or less. This benefit associated with AHF was, however, lost when the overall treatment time was extended to more than 10 weeks. In the CF group both locoregional control and survival were not influenced by the overall treatment time.

### Proliferation studies

The proliferation parameters (ploidy, T_s_ LI, and T_pot_) are comparable in the two treatment groups (Table 1). There were no significant differences between different clinicopathological categories as regards these parameters. (data not shown).

Overall, 44% (31 out of 70) of tumours were aneuploid. The proliferation parameters of diploid and aneuploid tumours did not seem to differ significantly (LI=11.5±5.4%, for diploid and 11.7±5.5% for aneuploid tumours, *P*=0.9, T_pot_=4.4±3.1 d for diploid and 5.1±3.1 d for aneuploid tumours, *P*=0.4).

Ploidy and the length of T_s_ did not seem to influence the treatment end-results (data not shown). Local control did not also correlate with the LI or T_pot_ but there was a significant correlation between T_pot_ and survival (*P*=0.05) ( Table 5

**Table 5:**
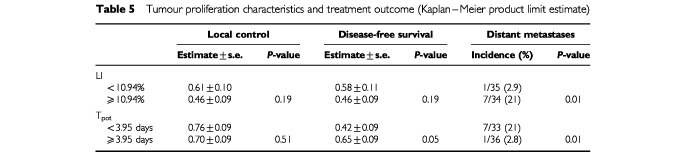
Tumour proliferation characteristics and treatment outcome (Kaplan–Meier product limit estimate)

). Applying the Cox's proportional hazards model, T_pot_ did not prove to be an independent prognostic factor after adjusting for the other clinicopathological and treatment factors (risk ratio of 0.95 with a 95% confidence interval of 0.78 and 1.09, *P*=0.49).

In an attempt to identify optimum cut-off points for different proliferation parameters that best predict treatment results the *corrected minimum P-value method* was applied (Altman *et al*, 1994). First the cut-off point was systematically varied to find out the points with the least *P* value (*P*_min_) according to the log rank test. This is then adjusted to find out a corrected *P*-value (*P*_cor_):


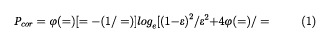


where φ denotes the probability density function and = is the (1-*P*_min_/2) quantile of the standard normal distribution. Applying this approach, a minimum *P*-value could be identified for locoregional control in relation to the LI and for survival in relation to the LI and T_pot_, but none of them attained a significant level (*P*⩽0.05) after correction using equation 1 ( Table 6

**Table 6:**
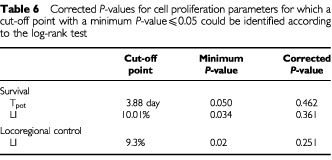
Corrected *P*-values for cell proliferation parameters for which a cut-off point with a minimum *P*-value⩽0.05 could be identified according to the log-rank test

).

Survival of patients with short T_pot_ did not significantly differ in the CF and AHF groups i.e. T_pot_ could not predict a survival benefit from AHF ( Table 7

**Table 7:**
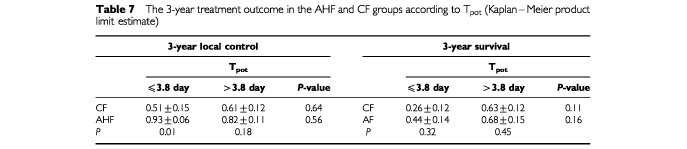
The 3-year treatment outcome in the AHF and CF groups according to T_pot_ (Kaplan–Meier product limit estimate)

). However, locoregional control was significantly better in the AHF than in the CF group in fast tumours (*P*=0.01). For slowly growing tumours both the tumour control and survival probabilities were not significantly different in the CF and AHF groups (Table 7).

Rapid tumour growth (indicated by a high LI and a short T_pot_) was associated with a significant increase in the incidence of distant metastases, *P*=0.01 (Table 5).

## DISCUSSION

According to current evidence-based concepts, postoperative radiotherapy of locally advanced HNC is still based on careful clinical observations (level IV evidence) rather than on randomized clinical trials (level I or II evidence). Search for an optimum postoperative radiotherapy dose in presence of high risk factors has been made. A randomized study identified an optimum dose of 57.5–63 Gy and demonstrated that a dose escalation beyond 63 Gy did not seem to improve the therapeutic ratio (Peters *et al*, 1993).

Compared with radical radiotherapy for HNC, very few studies tested the therapeutic advantages of accelerated radiotherapy in the postoperative radiotherapy of high-risk patients. A notable example is the conjoint prospective randomized study reported by Ang *et al* (1999) where a concomitant boost regimen (63 Gy in 5 weeks) was compared with CF to the same total dose but given over 7 weeks. Interim results pointed out a trend for better local control and survival at the expense of more severe acute reactions but without an apparent increase in late morbidity. For high risk patients, the present study demonstrated that AHF can significantly improve the locoregional control rate though without a significant survival advantage. The favourable influence of a short treatment time was further substantiated by the demonstration that the best locoregional control and disease-free survival rates were obtained in the AHF arm when the treatment was given within less than 6 weeks after surgery and when the overall treatment time did not exceed 10 weeks (Table 4). However, the therapeutic benefit of AHF relative to CF was masked when the surgery-radiotherapy gap exceeded 6 weeks and also when the overall treatment time was longer than 10 weeks. It seems therefore that the potential therapeutic benefit of an accelerated treatment can be counterbalanced by a long gap between surgery and radiotherapy. It is interesting to note that the surgery-radiotherapy gap and the overall treatment time did not seem to influence the outcome of treatment in the CF group. This suggests that the length of the actual radiation treatment time is more significant than the surgery-radiotherapy gap probably due to occurrence of an accelerated repopulation process during the actual postoperative radiation treatment time similar to that thought to occur during radical radiotherapy of HNC (Withers *et al*, 1988).

In case of radical radiotherapy of HNC, the influence of pretreatment tumour proliferation parameters on the treatment outcome is a controversial issue (Awwad *et al*, 1992; Lochin *et al*, 1992; Bourhis *et al*, 1993; Eschwege *et al*, 1997; Zackrisson *et al*, 1997; Høyer *et al*, 1998). A multicenter analysis of 476 patients in which 11 centres participating failed to demonstrate an association (Begg *et al*, 1999). Such negative results do not support the notion that T_pot_ can predict repopulation taking place during radical radiotherapy. Nevertheless, there is strong evidence that, in a radical radiotherapy setting, shortening of the treatment time improves the treatment outcome This is in keeping with the concept that repopulation is a strong determinant of this outcome (Horiot *et al*, 1997; Saunders *et al*, 1997; Withers *et al*, 1988). In the present postoperative radiotherapy study, we set out to test the potential value of tumour proliferation parameters to predict the overall treatment results. The possibility of their use as a guide for selecting patients for accelerated treatment was subsequently tested. Since FCM tends to underestimate the proliferative activity of diploid tumours (Bennett *et al*, 1992; Wilson, 1993; Høyer *et al*, 1998) it was combined with measurement of the LI using immunohistochemistry. Nevertheless, none of the proliferation parameters proved to be an *independent* predictor of treatment outcome. Despite lack of a definite association between T_pot_ and treatment outcome in the present postoperative radiotherapy setting, treatment acceleration was associated with a better locoregional control. As in radical radiotherapy, repopulation seems, therefore, to be an important determinant of treatment outcome in postoperative radiotherapy. This is further supported by the aforementioned influence of surgery-radiotherapy gap and the overall treatment time.

As expected, the treatment results of slow tumours did not significantly differ in the AHF and CF groups. AHF did not seem to provide a survival benefit to fast tumors but may offer a better local control (Table 7). This suggests the need for additional treatment (such as concomitant chemoradiotherapy) in order to improve survival of patients with fast growing tumours particularly since the risk of distant metastases was shown, in the present study, to be associated with a rapid tumour growth as indicated by a short T_pot_.

At present, the median values of the proliferation parameter are usually selected as cut-off points to identify risk groups. This approach is associated with a considerable loss of information. The minimum *P*-value approach has been proposed as a means of reducing (though not completely eliminating) the risk of missing a significant association (Altman *et al*, 1994). Since this approach is associated with a marked increase in type 1 error rate and may thus give false positive associations, a correction factor similar to that given by equation 1 has to be applied. In the present study none of the three cut-off points with minimum *P*-value ⩽0.05 according to the log rank test, proved to have a significant corrected *P*-value although minimum *P*-values as low as 0.027 are observed (Table 6). This is in accordance with the observation that none of the proliferation parameters proved to be an independent predictor of the treatment outcome when using standard statistical procedures based on the median values as cut-off points. The potential validity and use of such an approach is currently further tested using the clinical material of the present and other studies.

Compared with CF, AHF involves a faster rate of dose delivery, which might interfere with the regenerative response (Thames *et al*, 1990; Trotti *et al*, 1998). This can account for the early and rapid development of acute mucositis and its greater severity in case of treatment acceleration. However, the severity of early mucosal reactions did not generally interfere with completion of the prescribed treatment. The frequency of late reactions (fibrosis and oedema) is expected to be lower in the AHF group due to a smaller fraction size and total dose. However, the incidence of oedema and fibrosis was apparently higher in the AHF though the difference is not statistically significant. One interpretation would be that some late reactions are in fact consequential reactions that are driven mainly by mechanisms related to epithelial injury (Denham *et al*, 2000). Such reactions are then expected to be less influenced by the fraction size and to be influenced by the overall treatment time. Moreover, the surgical trauma might induce a proliferative response in the connective tissue and blood vessels and thus render their reactions more similar to that of early reacting tissues (Tibbs, 1997). All patients suffered from some degree of late xerostomia, which persisted for 2 years at least. There was a tendency, though statistically insignificant for xerostomia to be more severe in the AHF group (*P*=0.16). In view of the possibility that the volume of the parotid gland included within the high dose region can differ widely in different patients, this small difference is difficult to interpret on a radiobiological basis.

In conclusion treatment acceleration can provide better local control in the postoperative radiotherapy of locally advanced HNC suggesting that repopulation is an important determinant of treatment outcome. T_pot_ did not prove to be a good indicator of repopulation and this limits its use in selecting patients for AHF. To gain a full benefit from treatment acceleration, the surgery-radiotherapy gap and the overall treatment time should not exceed 6 and 10 weeks respectively.
